# *In vivo* studies investigating biodistribution of nanoparticle-encapsulated rhodamine B delivered *via* dissolving microneedles

**DOI:** 10.1016/j.jconrel.2017.04.022

**Published:** 2017-11-10

**Authors:** Joakim Kennedy, Eneko Larrañeta, Maelíosa T.C. McCrudden, Cian M. McCrudden, Aaron J. Brady, Steven J. Fallows, Helen O. McCarthy, Adrien Kissenpfennig, Ryan F. Donnelly

**Affiliations:** a School of Pharmacy, Queen's University Belfast, 97 Lisburn Road, Belfast BT9 7BL, UK; b Centre for Experimental Medicine, School of Medicine, Dentistry and Biomedical Sciences, Queen's University Belfast, University Road, Belfast BT9 7BL, UK

**Keywords:** Microneedles, Nanoparticles, Biodistribution

## Abstract

Nanoparticles (NPs) have undergone extensive investigation as drug delivery and targeting vehicles. NP delivery is often *via* the parenteral route, reliant on administration using hypodermic needles, which can be associated with patient compliance issues and safety concerns. In the recent past, the intradermal delivery of NPs, *via* novel dissolving microneedle (MN) arrays has garnered interest in the pharmaceutical community. However, published studies using this combinatorial approach have been limited, in that they have focussed on the use of *in vitro* and *ex vivo* models only. The current study was designed to answer the fundamental question of how such NPs are distributed in an *in vivo* murine model, following MN-mediated delivery. Rhodamine B (RhB) was employed as a model tracer dye to facilitate study of biodistribution. Following MN application, RhB was detected in the livers, kidneys, spleens and superficial parotid lymph nodes of the mice. Uptake into the lymphatics was of particular note, as it points towards the potential for utilisation of a minimally-invasive MN delivery strategy in controlled targeting of active drug substances and vaccines to the lymphatics. The use of such a delivery system could, following further development, have far-reaching benefits in enhancement of immunomodulatory and anti-cancer therapies. As a consequence, further investigation of MN/NP combinatorial delivery strategies is warranted.

## Graphical abstract

**Figure:**
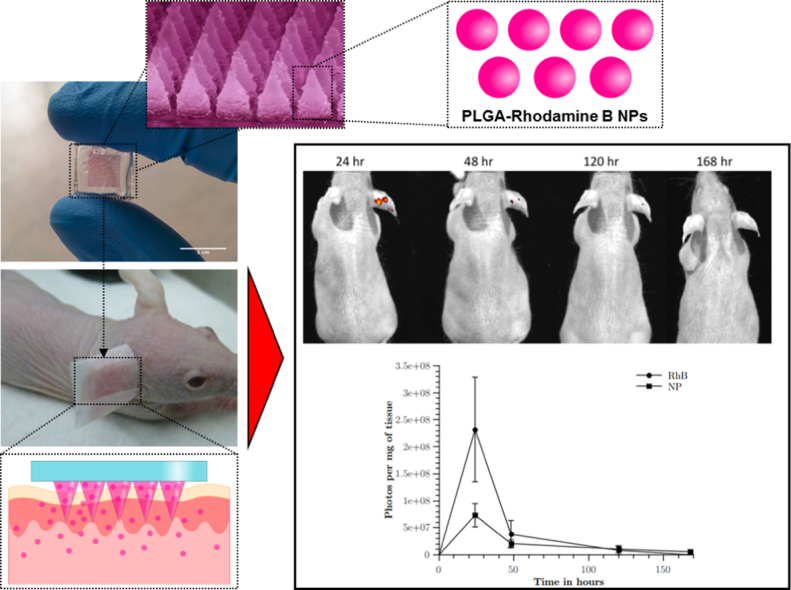

## 1 Introduction

Nanomedicine can be defined as the use of nanoscale or nanostructured materials in medicine, eliciting medicinal effects [1], [2]. The interest in this discipline has grown exponentially over the course of the last 25 years. One of the main areas of focus of nanomedicine is drug delivery. Nanoparticles (NPs) have been extensively used as vehicles to deliver drugs, vaccines, proteins and nucleotides [3]. As demonstrated in the literature, a wide variety of NP formulations have been fabricated using compounds such as lipids, polymers, sugars or metals, among many others [4]. NPs exhibit distinctive, size-dependent physico-chemical properties and present numerous advantages over conventional drug delivery systems [2], [5]. This mode of delivery provides protection for encapsulated cargo against proteolytic or chemical degradation and allows sustained drug release over prolonged periods of time [4]. In addition to these capabilities, NPs can also provide targeted drug delivery to certain parts of the body when modified with particular ligands [6].

The routes of administration for NP formulations are diverse and include intravenous, pulmonary, oral, nasal and ocular delivery [7]. Oral delivery is often the preferred route, but it presents several drawbacks, predominantly, drug degradation in the gastrointestinal tract and lack of NP absorption in the small intestine. In addition to this, first-pass metabolism can potentially destroy a drug before it can reach the systemic circulation [8], [9]. Consequently, the parenteral delivery route is viewed as a viable alternative to oral delivery. This route allows direct administration of nanomedicines into the bloodstream, or directly into a specific tissue, thus bypassing the aforementioned limitations associated with the gastrointestinal tract [10]. However, the parenteral route relies on administration using hypodermic needles, significantly reducing patient compliance, as it is often associated with pain [11]. Furthermore, this route of administration results in the generation of medical sharps waste, increasing the risk of disease transmission by needle re-use or needle-stick injury. This is of particular concern in countries in the developing world [12].

An alternative to these delivery strategies is *via* the transdermal delivery route. Transdermal delivery systems allow the administration of medicines in a non-invasive manner, potentially allowing self-administration. However, the barrier properties of the outermost layer of the skin, the *stratum corneum* (SC), limits the number of drugs that can be administered *via* this route to those with very specific physiochemical properties, most notably small size [13], [14]. Accordingly, passive permeation of NPs through this layer is extremely limited [4]. One possible means of improving NP administration may be through the use of microneedles (MNs). MNs are minimally-invasive devices that allow intradermal and transdermal administration of vaccines and drug substances by painless penetration of the SC [8], [15], [16], [17], [18]. MNs can be self-administered [19], [20] and, due to their unique ability to facilitate administration of drugs and vaccines across the skin, they have garnered much attention over the past decade [18], [19].

The intradermal delivery of NPs *via* MNs has undergone some rather limited investigation over the course of recent years, but the majority of the studies carried out to date have focused solely on *in vitro* and *ex vivo* experiments. Indeed, few studies have investigated MN/NP combinatorial delivery systems *in vivo* [2]. Accordingly, in the present exploratory study, we probed, for the first time, the biodistribution of a model water soluble compound following MN delivery. Both NP-encapsulated rhodamine B (RhB) and free-RhB were utilised in this work, so as to elucidate any differences. Specifically, the influences of using MN arrays containing RhB-loaded NPs (RhB/NP) in the needles and not the baseplate and MN arrays loaded with free-RhB were evaluated in an *in vivo* murine model.

## 2 Material and methods

### 2.1 Materials

Rhodamine B chloride, acetonitrile, methanol, poly(vinylpyrrolidone) (PVP) K90 and hydrochloric acid (HCl) were purchased from Sigma-Aldrich (Gillingham, Dorset, UK). Poly(lactic-*co*-glycolic acid) (PLGA) RG504H, was obtained from Boehringer Ingelheim GmbH (Ingelheim, Germany). Sodium hydroxide (NaOH) was obtained from VWR International (Lutterworth, Leicestershire, UK). PVP K29/30 was obtained from Ashland Inc. (Kidderminster, UK).

### 2.2 Fabrication of biodegradable PLGA nanoparticles

The NPs were fabricated by nanoprecipitation, also termed solvent displacement, as documented previously [21], [22]. The method was adapted and modified from a previous publication [23]. In brief, for each batch of NPs, 10 mg PLGA (RG504H) was dissolved in 9.7 ml of acetone containing 0.3 ml of 0.1 mg ml^− 1^ RhB. The PLGA/acetone mixture was then added to 10 ml of water and stirred at 800 rpm (rpm) until the acetone evaporated. Once the NPs were formed, the suspension was dialysed for two days using dialysis membrane with molecular weight cut-off of 14 kDa, to facilitate removal of any unbound RhB. PVP (58 kDa) was then added to the nanosuspension as cryoprotectant in a ratio of PVP to NPs, 2:1. The cryoprotectant was mixed into the suspension by adding the desired quantity of polymer and allowing the suspension to mix for at least 30 min to ensure the cryoprotectant had completely dissolved prior to freeze-drying. PLGA NPs were then dried using the freeze-drying cycle outlined in Table 1. The resulting NPs were characterised using a Zetasizer Nano ZS (Malvern Instruments Malvern, Worcestershire, UK).

**Table 1:** The freeze-drying cycle for NP drying.

Step	Temperature (°C)	Pressure (torr)	Time (min)	Type of step
1	5	760	10	Hold
2	− 40	760	60	Ramp
3	− 35	190	180	Hold
4	− 30	190	60	Ramp
5	− 30	190	180	Hold
6	− 25	190	60	Ramp
7	− 25	190	180	Hold
8	25	190	120	Ramp
9	25	190	360	Hold
10	25	50	600	Hold

### 2.3 Determination of the amount of RhB encapsulated in the NPs

To determine the amount of RhB encapsulated in PLGA NPs, three samples of known mass were taken from freeze dried powder samples. These samples were re-suspended in a known volume of 1 M NaOH and left overnight in order to dissolve the PLGA NPs. Following 24 h, the solutions were neutralised with 1 M HCl. The neutralised solutions were diluted two-fold with high performance liquid chromatography (HPLC) grade water. HPLC analysis was carried out on an Agilent Technologies 1200 Series system, with an auto sampler, binary pump, degasser and a fluorescence detector (Excitation: 546 nm; Emission: 570 nm). A Waters Symmetry 300™ C4 column thermostatically controlled at 25 °C was utilised. The mobile phase consisted of a mix of HPLC grade water, pH 7:organic mixture (80% methanol; 20% acetonitrile), in the ratio 45:55 (v/v). The injection volume was 20 μl, while the flow rate was 1 ml min^− 1^.

### 2.4 Fabrication of dissolving MNs

**Fig. 1:**
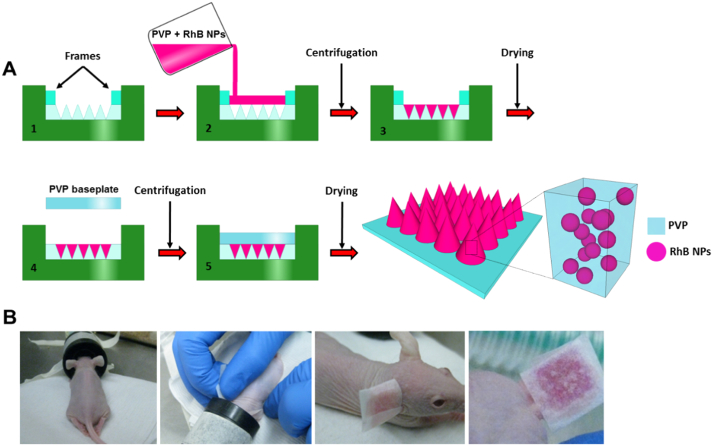
Schematic representation of the fabrication process of the dissolving MN arrays (A). Digital photographs of the MN application process to the dorsal surface of mouse ears *in vivo* (B).

The MN were fabricated by primary casting of a NP-containing formulation into the needles of the MN arrays only, with a very shallow, cargo-less baseplate behind the needles. The moulds employed in the study consisted of a 19 × 19 array with needle heights of 600 μm, width at base of 300 μm and interspacing at base of 50 μm in a mould area of 0.5 cm^2^. The MN fabrication methodology was modified from previously published studies [24], [25]. Initially, a silicone frame was designed and affixed into pre-manufactured silicone MN moulds [24] using a small amount of PVP-gel on the underside of the frames. These frames were then dried into the moulds at room temperature for 45 min. The framed-moulds were then used to cast NP-containing formulation into the needles only. To achieve this, a quantity of primary casting gel (30% (w/w) 58 kDa PVP aqueous solution, with an equal mass of the freeze dried NPs), sufficient to cover the array, was added into the framed-mould and spread within the framed area using a spatula. The moulds were then centrifuged for 10 min at 3500 rpm. After centrifugation, the frames were removed from the moulds and the arrays were dried at room temperature for 1.5 h. Post-drying, a pre-formed baseplate, consisting of 15% (w/w) 360 kDa PVP, was placed behind the needles cast into the moulds using 10 μl of 50% (w/w) 360 kDa PVP as an adhesive. The moulds were then centrifuged at 5000 rpm for 90 min and the arrays were dried overnight at room temperature. Control MN arrays containing equivalent amounts of free-RhB were also prepared. A schematic representation of this methodology is presented in Fig. 1A.

### 2.5 Application of MN arrays

The transdermal delivery of RhB-loaded NPs and free-RhB using dissolving MN arrays was evaluated using hairless mice (strain SKH1-Hrhr). All animal experiments throughout this study were conducted according to the policy of the Federation of European Laboratory Animal Science Associations and the European Convention for the protection of vertebrate animals used for experimental and other scientific purposes, with implementation of the principles of the 3R's (replacement, reduction, refinement). Ethical permission for the experiments was obtained from the Queen's University Belfast, Biological Services Unit (BSU) and the work was carried out under Project Licence 2794, as granted by the UK Home Office. Mice employed in the study were aged between 14 and 18 weeks and both males and females were used. Mice were anaesthetised using gaseous anaesthesia (2–4% isoflurane in oxygen) and MNs were inserted, with pressure applied for 5 min, to the dorsal surface of the right ear and kept in place for 24 h with 3M™ Micropore™ medical tape (3M™, St. Paul, MN, USA) cut into a T-shape. The application process is documented in Fig. 1B. All MN arrays were removed after 24 h.

### 2.6 In vivo visualisation of NP-derived RhB and free-RhB using IVISR imaging tool

In order to visualise the uptake of RhB-loaded NPs or free-RhB over time, an *in vivo* imaging system (IVISR) was utilised (Xenogen IVISR Imaging System 200 Series, Caliper LifeSciences, Hopkinton, MA). In total, twelve mice were imaged at four predetermined time intervals, namely: 24, 48, 120 and 168 h post-application. After seven days of imaging, all mice were euthanised and superficial parotid lymph nodes, spleens, livers, kidneys, and right ears were all excised and imaged.

Specifically, the mice were split into four groups: (i) three males were treated with RhB-loaded NP incorporated into MN (RhB/NP MNs); (ii) three males were treated with free-RhB incorporated into MN (free-RhB MNs); (iii) three females treated with RhB/NP MNs and (iv) three females treated with free-RhB MNs. This identification terminology was subsequently used throughout the study.

### 2.7 Quantification of RhB localised in mouse organs

The fluorescence intensity of the NP-delivered or free-RhB was also measured using IVISR. Utilising 21 male and 21 female mice, three cohorts, consisting of seven mice each, were assembled, namely: 24, 48 and 120 h. In all instances, six mice had MN applied to the right ear and one mouse, an untreated control, was also included in the cohort. Half of the males and half of the females in each cohort had RhB/NP MNs applied and the other half had free-RhB MNs applied. At the pre-determined experimental end points, the mice were euthanised and the superficial parotid lymph nodes, spleens, livers, kidneys and right ears were removed, weighed and imaged. Following removal of MN from the ears, the area was cleaned with tissue paper soaked in 70% (v/v) alcohol so as to prevent superficial tracer dye causing overestimation in fluorescence signals. For biodistribution studies, the fluorescence reading was corrected by subtracting the mean readings from the control tissue and normalised by dividing with the tissue mass, as outlined by Merkel et al. [26].

## 3 Results

The mean particle size of RhB-loaded NPs in a representative batch was determined to be 69.3 ± 4.6 nm, with a polydispersity index of 0.27 ± 0.03, n = 6.

Dissolving MN arrays were then fabricated in such a way as to maximize the amount of RhB loaded into each array. Upon preparation of the RhB/NP or free-RhB casting gels, the mean concentrations (ng mg^− 1^) and amounts (μg) of RhB in the representative gels and in the subsequent MN arrays produced were determined, with data presented in Table 2.

**Table 2:** Concentrations (ng mg^− 1^) and amounts (μg) RhB in the casting gels and MN arrays, respectively. The values are given as the mean ± standard error, n = 6.

Formulation	Cargo	[RhB] (ng mg^− 1^) in casting gel	RhB (μg) per MN array
RhB/NP MNs	NP encapsulated RhB	226 ± 13	7.8 ± 0.3
Free-RhB MNs	Free-RhB	208	11.7 ± 0.6

**Fig. 2:**
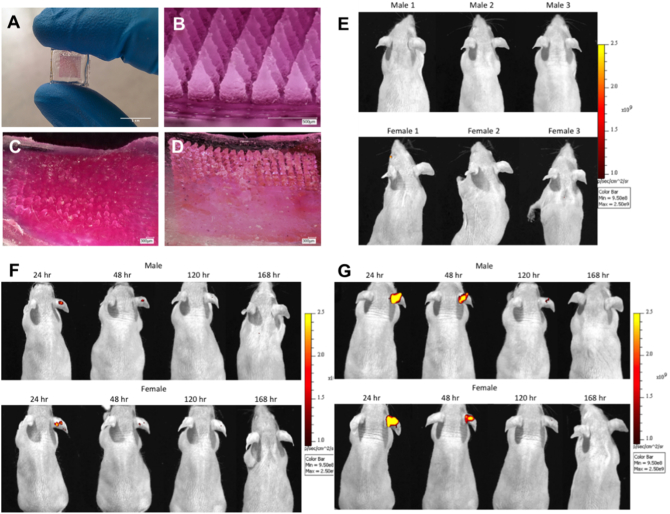
Digital images of the dissolving RhB/NP MN arrays before insertion into mouse ears (A and B). Micrographs of MN arrays loaded with RhB/NPs (C) and free-RhB (D), post-24 h application to mouse ears. IVISR images of three male and three female mice from the control treatment cohorts (*i.e.* no MN applied to the ears) (E). Fluorescence detected at different time points (24, 48, 120 or 168 h), post-MN application in test mice from the RhB/NP MN cohort: male (upper panel) and female (lower panel) mice captured using the IVISR system (F). Fluorescence detected at different time points (24, 48, 120 or 168 h), post-MN application in test mice from the free-RhB MN cohort: male (upper panel) and female (lower panel) mice captured using the IVISR system (G). In all instances, the colour indicates how many photons were detected.

Fig 2A and B shows representative images of the dissolving MN arrays containing RhB/NPs which were localised into the needles themselves.

The MN arrays were successfully inserted into the ears of the mice and held in place for 24 h. They were then removed and the remnants of the MNs were imaged using a light microscope (VHX-700F digital microscope, Keyence, Milton Keynes, UK). As illustrated in the representative images presented in Fig. 2C and D, the vast majority of needles on the arrays dissolved following application to mouse ears for 24 h.

The fluorescence intensity of the NP-delivered and free-RhB delivered into the ears using MNs were visualised using the IVISR system. IVISR images of three male and three female control mice from the treatment cohorts are presented in Fig. 2E. As these mice received no MN treatment, there is consequently no evidence of RhB delivery to the ears of these mice. To study the varying drainage of RhB/NPs and free-RhB from the ears of treated mice, images of a single representative male or female mouse from each of the two treatment cohorts, taken at pre-determined time intervals (24, 48, 120 or 168 h), are presented in Fig. 2F (RhB/NP cohort) and 2G (free-RhB cohort). Comparison of the images presented in the two figure panels makes it obvious that more RhB was delivered into the ears of the mice when MN arrays containing free tracer dye (free-RhB) were used rather than RhB/NP MN arrays. In both delivery approaches, the signal decreased over time. This suggests that, in both instances, the free-RhB or RhB/NPs migrated from the ear and did not accumulate there. After seven days, the signal returned to baseline levels in all mice tested.

**Fig. 3:**
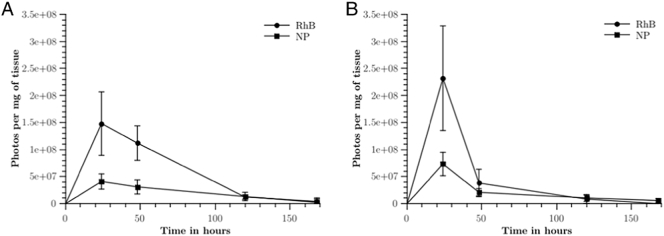
The normalised concentrations of photons per mass of tissue in the excised ears of male (A) and female (B) mice, plotted as a function of time (RhB = free-RhB; NP = RhB/NP). The values presented are the means ± standard error, n = 3.

The IVISR system captures images from above, imaging the transverse plane of the subject. Therefore, it is not possible to obtain an accurate fluorescent intensity reading using this image plane. To record more accurate readings, mice were culled at pre-determined time points and the ears to which the MN arrays were applied were removed and imaged along the coronal plane. The number of photons recorded was divided by the mass of the excised ear, to produce a “*concentration*” of photons per mass of tissue, as previously outlined by others [26]. The concentrations of photons per mass of tissue for the two treatment cohorts (free-RhB and RhB/NP) are presented in Fig. 3A and B. In the cases of both male and female mice, the greatest concentrations of free-RhB and NP derived RhB were present in excised ears 24 h post-MN application. It is evident that after 120 h, the free-RhB and NP-derived RhB have migrated away from the ear as the concentrations have markedly decreased. Comparing between the two genders, it appears that the uptake in the females is faster than the males for both free-RhB and NP-derived RhB, as the concentration difference between 24 h and 48 h post-application is greater, showing a steeper change in the curve. Other properties of the curves are recorded in Table 3.

**Table 3:** Data obtained from the curves for free-RhB and RhB/NP in mice ears. AUC was calculated from the mean curve. Values are given as means ± standard error, n = 3.

Sex/formulation	*t*_*max*_ (h)	*C*_*max*_/10^7^ (photons/mg)	AUC
Male/RhB/NP	24	4.1 ± 1.4	3.31 × 10^9^
Male/free-RhB	24	14.8 ± 5.9	9.76 × 10^9^
Female/RhB/NP	24	7.3 ± 2.2	3.53 × 10^9^
Female/free-RhB	24	23.2 ± 9.7	7.89 × 10^7^

**Fig. 4:**
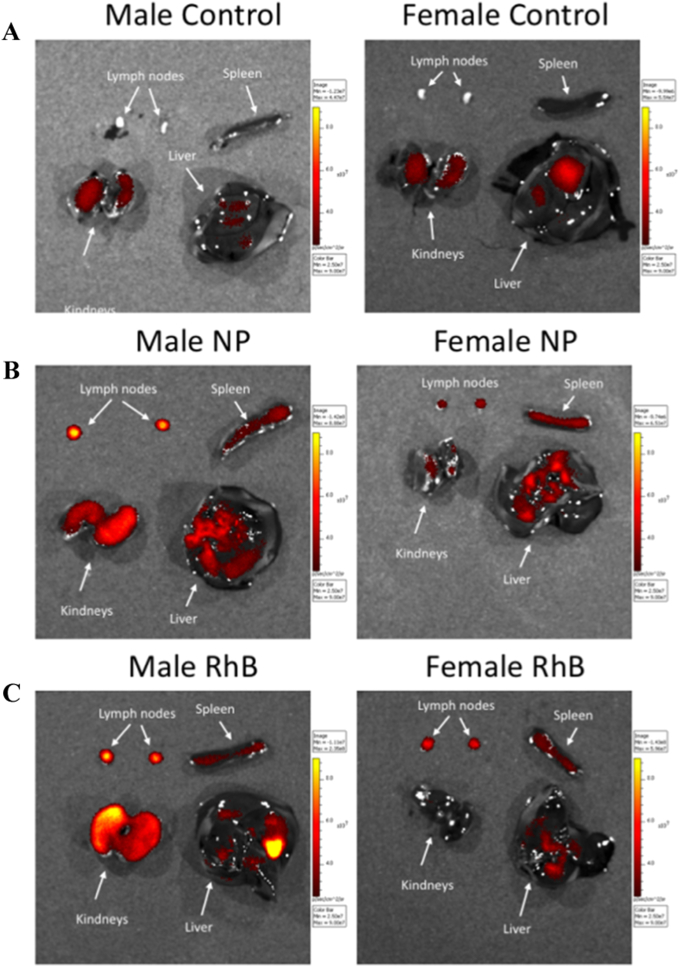
Sample IVISR images of parotid lymph nodes, spleens, livers and kidneys removed from control (no MN treatment) male and female mice (A), RhB/NP (NP) treated male and female mice (B) and free-RhB (RhB) treated male and female mice (C). All images are from mice which were culled 48 h post-MN treatment.

In order to determine which organs the RhB was delivered to, mice were culled 48 h post-MN application and specific organs were excised. These were placed on a dark sheet and imaged using the IVISR with sample images presented in Fig. 4. The fluorescence measured was then used to determine the biodistribution of RhB in the mice.

**Fig. 5:**
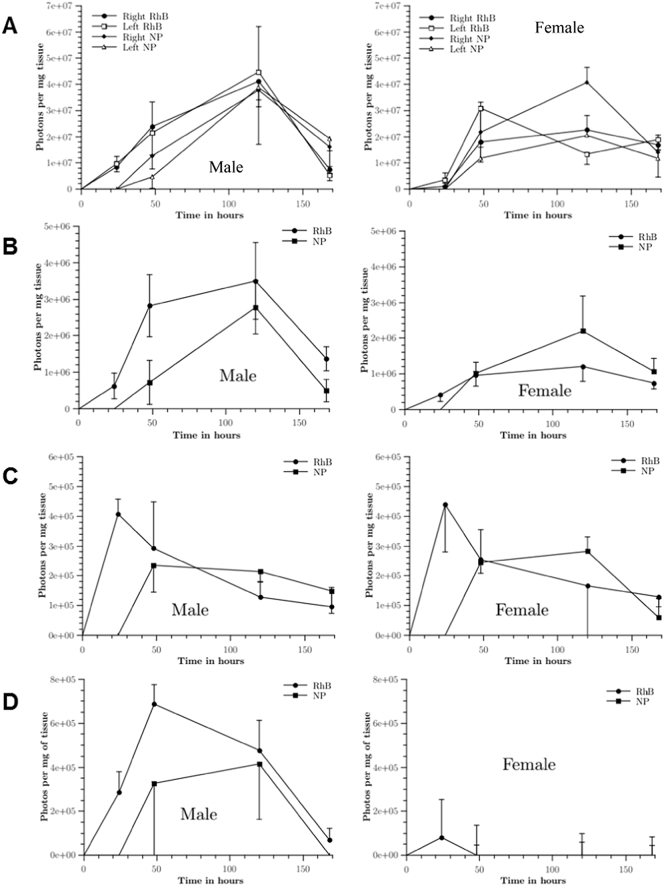
The normalised concentrations of photons, indicative of free-RhB (RhB) or NP-derived RhB (NP) delivery per mass of tissue in the superficial parotid lymph nodes (A), spleens (B), livers (C) and kidneys (D) of male or female mice, plotted as a function of time. The values shown are the means ± standard error, n = 6.

The free-RhB and RhB/NPs delivered into the ears may be taken up either by the blood or lymph capillaries. If taken up by the lymph capillaries, it would drain to the nearest lymph node, the superficial parotid lymph node. RhB/NPs may also be taken up by antigen-presenting cells in the viable skin layers, which then migrate to the draining lymph node. This is less likely to happen for free RhB. To assess the degree of lymphatic uptake of the free-RhB or NP-derived RhB, the superficial parotid lymph nodes were extracted (Fig. 4). As the lymphatic circulatory system is one unified system, the concentration in the whole lymphatic system should reach a uniform concentration level [27]. As a result, the free-RhB and NP-derived RhB should be detectable in both the left and right (same side as MN application) superficial parotid lymph nodes. Consequently, the concentration of RhB in the left superficial parotid lymph node can be used to determine when the levels of free-RhB and NP-derived RhB in the lymphatic circulatory system have reached a steady state. The concentration of free-RhB and NP-derived RhB in the superficial parotid lymph nodes are graphically presented, as a function of time, in Fig. 5A. The concentrations of free-RhB and NP-derived RhB in spleens, livers and kidneys are then presented in Fig. 5B, C and D, respectively.

As depicted in Fig. 5A, the time taken for the NP-derived RhB to reach detectable levels in the lymph nodes exceeded 24 h. Also, the concentration in the lymphatic circulatory system had not reached a steady state, even 48 h post-application. The concentration of NP-derived RhB in the right lymph nodes was consistently higher than that detected in the left lymph nodes, with the highest concentration of NP-derived RhB in the lymph nodes recorded 120 h post-application. It was not possible to determine whether this was the maximal concentration, however, as it is possible a maximal concentration had been reached between the 48 and 120 h sampling times. Seven days (168 h) post-application, the fluorescence readings returned almost to basal levels. Male mice appeared to clear free-RhB faster than the NP-derived RhB, while female mice exhibited very similar concentrations of free-RhB and NP-derived RhB in the lymph nodes up to 168 h post-application. The NP-derived RhB concentrations in the lymph nodes one week post-MN application were approximately the same in both male and female mice. Pharmacokinetic properties were calculated from the curves generated in Fig. 5A and are summarised in Table 4.

**Table 4:** Properties of concentration *versus* time curves for free-RhB and RhB/NP in either left (L) or right (R) superficial parotid lymph nodes. AUC was calculated from the mean curve. Values given are means ± standard error, n = 3.

Sex/formulation	L/R	*t*_*max*_ (h)	*C*_*max*_/10^7^ (photons/mg)	AUC/10^9^
Male/RhB/NP	L	120	3.9 ± 2.2	3.01
Male/RhB/NP	R	120	3.8 ± 0.4	3.26
Male/free-RhB	L	120	4.5 ± 1.7	4.08
Male/free-RhB	R	120	4.1 ± 0.9	4.00
Female/RhB/NP	L	120	2.1 ± 0.7	2.08
Female/RhB/NP	R	120	4.1 ± 0.5	3.82
Female/free-RhB	L	48	3.1 ± 1.3	2.82
Female/free-RhB	R	120	2.3 ± 0.2	2.65

Another organ of the lymphatic system is the spleen. The concentrations of free-RhB and NP-derived RhB in the spleens of treated mice, as a function of time, are presented in Fig. 5B and the summary of the properties determined from this graph are displayed in Table 5. Irrespective of delivery vehicle, the concentrations of RhB in the spleens was approximately an order of magnitude lower than that recorded in the superficial parotid lymph nodes. For those mice treated with free-RhB loaded MN arrays, RhB was detected in the spleen 24 h post-application, while those mice treated with RhB/NP loaded MN arrays did not have any detectable concentrations of the molecule until 48 h post-application.

**Table 5:** Properties of the concentration *versus* time curves for free-RhB and RhB/NP in mouse spleens. AUC was calculated from the curve of concentration *vs.* time. Values given are mean ± standard error, n = 3.

Sex/formulation	*t*_*max*_ (h)	*C*_*max*_/10^6^ (photons/mg)	AUC/10^8^
Male/RhB/NP	120	2.8 ± 0.7	2.13
Male/free-RhB	120	3.5 ± 1.1	3.93
Female/RhB/NP	120	2.2 ± 1.0	2.06
Female/free-RhB	120	1.2 ± 0.4	1.46

The third organ investigated was the liver. With its rich blood supply, and the fact that most of the detoxification in the body occurs in the liver, the concentration of free-RhB and NP-derived RhB in the liver can be used to profile systemic exposure to this molecule. The concentration of RhB measured in the livers of treated mice, as a function of time, is presented in Fig. 5C and the summary of the properties calculated from this graph are displayed in Table 6. As depicted in Fig. 5C, free-RhB and NP-derived RhB have different concentration profiles in this organ. The free-RhB profile is indicative of the normal pharmacokinetics of a drug injected subcutaneously; firstly, a rapid absorption phase with a maximal concentration recorded, followed by a slower elimination phase. This is compared to the profile for the NP-derived RhB, where the concentration increased to a maximal level and then remained constant up to five days' post-application, when the concentration then decreased. In the case of NP-derived RhB, there were no detectable levels of RhB in the livers until 48 h post-application. The concentration of the molecule remained relatively constant after the 48 h and it was slowly eliminated thereafter. Comparing the area under the curve (AUC) and the maximal concentration of RhB, C_max_, in order to compare between the two MN application methods, it can be seen that both values are lower in the case of the RhB/NP MNs. However, direct comparison cannot be made, as the two types of MNs presented slightly different RhB loadings (Table 2).

**Table 6:** Properties of the concentration *versus* time curves for free-RhB and RhB/NP in mouse livers. AUC was calculated from the curve of concentration *vs.* time. Values given are mean ± standard error, n = 3.

Sex/formulation	*t*_*max*_ (h)	*C*_*max*_/10^5^ (photons/mg)	AUC/10^7^
Male/RhB/NP	48	2.4 ± 0.9	2.13
Male/free-RhB	24	4.1 ± 0.5	3.93
Female/RhB/NP	120	2.8 ± 0.5	2.06
Female/free-RhB	24	4.4 ± 1.6	1.46

The kidney is responsible for the filtering and excretion of unwanted materials from the body. Thus, as RhB is being excreted, it passes through the kidneys. As a result, the photons measured in the kidneys are indicative of RhB which is possibly being prepared for excretion. The concentration profile of RhB in the kidneys is graphically presented in Fig. 5D. The concentration profiles of males and females were markedly different. In females, detectable RhB was only recorded at 24 h from free-RhB-loaded MN. It is not clear why no other detectable RhB was recorded. Males treated with free-RhB MNs displayed faster accumulation of RhB and also a larger C_max_, as can be seen in Fig. 5D and presented in Table 7. The maximal concentration of RhB measured for the free-RhB cohort was at 48 h post-application, while the RhB/NP cohort displayed a maximal concentration in the kidneys at 120 h post-application.

**Table 7:** Properties of the concentration *versus* time curves for free-RhB and RhB/NP in mouse kidneys. AUC was calculated from the curve of concentration *vs.* time. Values given are mean ± standard error, n = 3.

Sex/formulation	*t*_*max*_ (h)	*C*_*max*_/10^5^ (photons/mg)	AUC/10^7^
Male/RhB/NP	120	4.2 ± 2.5	4.05
Male/free-RhB	48	6.9 ± 0.9	7.00
Female/RhB/NP	–	–	–
Female/free-RhB	24	0.8 ± 1.7	0.19

## 4 Discussion

Here we have shown, for the first time, that NPs delivered into the viable skin layers using MNs can be absorbed and distributed throughout the body.

When the MNs were applied to the ears of mice and held *in situ* for 24 h, the majority of the needles on the arrays dissolved. Following visual inspection of the MNs, RhB delivery into the mice was confirmed by carrying out *in vivo* and *ex vivo* imaging using an IVISR image system. NPs delivered using MNs appeared in the lymphatic system at a slightly slower rate than free RhB and free RhB appeared in the kidneys more quickly than the NP-bound dye. Biodistribution in other organs (spleen, liver) indicated higher RhB-associated fluorescence in the groups treated with free-RhB MNs. Biodistribution in female mice should distinct differences with that in males for both free and NP-bound dye. The reasons for this were not apparent, since the mice had similar weights and levels of physical activity, regardless of gender or formulation applied, including untreated controls.

In general, small drug molecules (< 1 kDa) are thought to be preferentially absorbed by the blood capillaries, due to their largely unrestricted permeability across the vascular endothelium, together with the high rate of filtration and reabsorption of fluid across the vascular capillaries (in the range of 20–40 l/day, in comparison to approximately 2–4 l/day of fluid drained by the lymph). A small molecule such as RhB should, therefore, predominantly be taken up by the blood capillaries of the dermal microcirculation, even though it has appreciable water solubility. In contrast, NPs which are too large (limits < 10 nm in size, or approximately 16–20 kDa for proteins) to be taken up by the blood capillaries should only be taken up by the lymphatics (10–100 nm for particles and 20–30 kDa for proteins) [28]. That RhB was found in the left ear lymph node, as well as that on the right and in distant organs following MN delivery of RhB NPs confirms that these particles were absorbed into the lymphatic system and then entered the systemic circulation. The removal of unbound RhB from the particles during formulation strongly suggests that the biodistribution observed is due to the particles themselves and not to released RhB.

Following initial delivery of RhB into the ears of the mice *via* either free-RhB MN or RhB/NP MN, the fluorescence signal at the delivery site became weaker over time, indicating that the free-RhB and NP-bound RhB were both migrating away from the ear. The elucidation of the exact mechanism by which this occurs is worthy of further investigation. The stronger fluorescence signal elicited following application of free-RhB MN suggests that a greater amount of RhB was delivered into those ears than when RhB/NP MN were employed. This was despite the fact that the MN arrays essentially had the same amount of RhB loaded into the needles.

A fluorescence signal from RhB/NPs was still detected at the injection site 48 h post-application. This supports previously-reported findings, which have documented that draining to the lymphatics is slower in certain regions of the body and that it can take up to 48 h for NPs to be detected in the draining lymph nodes [29]. The lymphatic circulatory system is a unidirectional circulatory system without a heart to pump the lymph around. Instead, the fluid is moved around through muscle movements. This explains why lymphatic uptake is much faster for injections in the limbs than in the ears, for example. Another study, which injected NPs subcutaneously into the footpad of rats, recovered 15% of the injected NP in the draining lymph node after 24 h, while 65% remained in the footpad [23]. This can be compared to the results in the present study, where no detectable levels of RhB in the draining lymph node were achieved 24 h post-application of RhB/NP MNs, proving that the draining from the ear and subsequent lymphatic uptake of the RhB/NP is relatively slow. Indeed, it has previously been shown that free-RhB injected subcutaneously completely migrated away from the injection site within 24 h, compared to RhB attached to polymeric surfactants, which was still present at the injection site 72 h post-injection [30]. In the same study, free-RhB was detected in the urine of the mice one to four hours after the injection. The free RhB, therefore, entered the systemic circulation quickly and was excreted by the mice. In the present study, it is clear that free-RhB enters the lymphatic system quite quickly. It is likely that at least some of the delivered dose is passively drained into the draining lymphatic adjacent to the application site. Some free-RhB may also enter the lymphatic system from the plasma, having previously been absorbed by the dermal microcirculation. This is in contrast to the NP-bound dye, which is likely to predominantly rely on slow drainage of interstitial fluid to carry it to the lymphatics for uptake. However, work previously carried out in our Group showed that fluorescently-labelled antigen-decorated NPs were taken up by professional antigen-presenting cells in the viable skin of mice *in vivo* and trafficked to the draining lymph nodes [31]. This process took approximately 48 h. If the PLGA used to make the NPs in the present study contained any lipopolysaccharide, it is possible that some of the NPs could have been carried slowly to the draining lymph node following cellular uptake,

The photon concentration profiles in the organs studied (spleen, liver, kidneys) all showed a delay in appearance for the NP-bound RhB, relative to the free dye. The peak fluorescence was also lower for the NP-bound dye. With the exception of the kidneys of female rats, where it didn't appear at all, the fluorescence due to the NP-bound RhB took longer to clear that due to the free dye. While the data overall may be influenced to some extent by the slightly lower RhB loading in the NP-laden MNs (Table 2) and the possibility of somewhat quenched fluorescence in the NPs, delayed appearance in and clearance from organs is most likely to be due to the distinctly difference biodistribution of the NP-bound dye. This is likely to be at least partly because it took longer for the RhB/NPs to enter the systemic circulation relative to free dye. Since the NPs are too large to be taken up by the blood capillaries, they must have entered the lymph vessels in the skin and migrated though the lymphatic system before they could enter the systemic circulation. Indeed, while the spleen forms an integral part of the lymphatic system, it only has efferent lymph vessels. This means it is only supplied by the cardiovascular system and, as a result, the NP-associated RhB fluorescence detected in the spleen here must have come from the general circulation.

It is likely that the RhB/NPs will diffuse more slowly through the dermal tissue than the free RhB molecules, which consequently means it will take longer for the RhB/NPs to migrate away from the application site than the free-RhB molecules. This phenomenon has been reported in a previous study, which compared the uptake of a free tracer with that of NPs following subcutaneous injection [32]. In Fig. 3, it can be seen that the photon concentration of the free-RhB is higher at the application site compared to that at the application site of the RhB/NPs but, in line with other published studies, the photon concentration decrease over time is faster, indicating that the draining of free-RhB is faster than that of RhB/NPs.

Biodistribution in the regional lymph nodes, liver, spleens, and kidneys is known to be dependent upon the injection/application site [32]. Since lymph is moving towards the base of the neck, where it flows into the subclavian veins and enters the cardiovascular system, it is expected that injection/application sites closer to the neck will result in a shorter distribution time for the NPs, with more of the NPs entering general circulation in the cardiovascular system. This is what was observed in the present study, complementary to that reported by others previously which found that smaller particles which had a higher lymphatic uptake also travelled faster through the lymphatic circulatory system and entered general circulation, resulting in a higher blood concentration and liver uptake [23], [33]. This is in contrast to the results generated when particulates were injected subcutaneously into mice paws where twice the amount of NPs were recovered in the lymph nodes, compared to the liver and blood, 24 h post-injection [34]. Using these previously documented studies and the results presented here, it can be concluded that the NPs are being taken up by the lymphatic circulatory system, but are also travelling through the lymphatics and entering the systemic circulation. Importantly, the experimental approach undertaken in the present study is likely to have influenced the kinetics, and possibly also the extent, of the observed biodistribution, since the MN application site used here is relatively close to the subclavian veins where the lymph drains.

It is clear that, in the present study, the MN-delivered NPs entered the lymphatic system, but did not remain there. However, there are numerous medical applications where lymphatic targeting and retention would be advantageous. These include not only vaccination and immunomodulation, but also enhanced treatment of tumours of the lymphatic system and management of metastases of solid tumours, which typically occurs through the lymphatics. If vaccines and therapeutic agents could be dosed mostly to their site of action in the lymphatics, therapeutic efficacy would most likely be increased and side-effects reduced. The retention of particulates, including NPs, in the lymph is mainly due to phagocytosis by macrophages [35]. As the use of smaller particles results in greater lymphatic uptake, but employing larger particles leads to more efficient lymphatic retention, a range of pharmaceutical interventions have been developed in a bid to increase lymphatic retention of drug molecules, including surface modifications, such as addition of branched polymers to NP surfaces [23], [34], [36].

## 5 Conclusion

In this exploratory study we showed, for the first time, that NPs delivered intradermally using dissolving MNs entered the lymphatic system. MN delivery, therefore, shows clear potential as a minimally-invasive alternative to subcutaneous delivery of nanoparticulate carriers intended for vaccination purposes or management of diseases of the lymphatic system. However, we were not able to control retention in the lymphatics. This is unsurprising, given that the particles employed were not specifically modified to prevent subsequent biodistribution *via* the systemic circulation. Since such work has never before been done, further understanding must now be developed. For example, we employed a single particle type with fixed size and charge here. Modulation of these parameters, along with alteration of the polymer matrix to control lipophilicity may well have profound effects on lymphatic uptake and retention. Similarly, MN application site is also likely to influence uptake and retention kinetics. Now that we have proved that MN-delivered NPs can enter the lymphatics, we have embarked upon a comprehensive programme of work to enhance retention and maximize dosing efficiency.
